# Executive Functioning in Chinese Patients With Obsessive Compulsive Disorder

**DOI:** 10.3389/fpsyt.2021.662449

**Published:** 2021-08-25

**Authors:** Huicong Ren, Haibin Li, Jin Huang, Nan Zhang, Ruiqin Chen, Wenjuan Liu, Zhaohui Zhang, Chencheng Zhang

**Affiliations:** ^1^The Second Department of Psychiatry, The Second Affiliated Hospital of Xinxiang Medical University, Xinxiang, China; ^2^Department of Psychological Medicine, Zhongshan Hospital, Fudan University, Shanghai, China; ^3^School of Public Health, Shanghai Jiao Tong University School of Medicine, Shanghai, China; ^4^Research Center of Brain and Cognitive Neuroscience, Liaoning Normal University, Dalian, China; ^5^Research Integrative Neuroscience, Deanery of Biomedical Sciences, University of Edinburgh, Edinburgh, United Kingdom; ^6^Departments of Psychiatry, The First Affiliated Hospital of Xinxiang Medical University, Xinxiang, China; ^7^Department of Neurosurgery, Center for Functional Neurosurgery, Ruijin Hospital, Shanghai Jiao Tong University School of Medicine, Shanghai, China; ^8^Shanghai Research Center for Brain Science and Brain-Inspired Intelligence, Shanghai, China

**Keywords:** obsessive-compulsive disorder, cognitive functions, CANTAB, executive function, yale-brown obsessive compulsive scale

## Abstract

**Introduction:** Studies have shown that patients with obsessive compulsive disorder (OCD) often perform more poorly than healthy control (HC) participants on cognitive tasks involving executive functions. Most studies, however, have been performed in Western countries and societies, making it uncertain whether impaired executive functions can also be observed among non-Western patients with OCD. To address this gap in the literature, we evaluated several executive functions in Chinese patients with OCD and HCs.

**Methods:** Participants included consisted of 46 Chinese patients with OCD (25 men, 21 women), ranging in age from 19 to 56 years, and 45 matched HCs without any self-reported lifetime psychiatric disorder. They all lived in Shanghai or the surrounding area. Five tests of the Cambridge Neuropsychological Test Automated Battery (CANTAB) were used to evaluate several executive functions (response inhibition, spatial working memory, planning, and cognitive flexibility) along with testing basic learning and visual recognition memory. Statistical tests using a Bonferroni-corrected significance level of p = 0.003 were performed to assess overall patient-control group differences in cognitive performance. Additionally, we explored performance differences between patients classified as having either relatively mild symptoms or severe symptoms based on the individual total scores on the Yale-Brown Obsessive-Compulsive Scale.

**Results:** There were no significant performance differences between patients with OCD and HC in any of the cognitive tests. Similarly, cognitive performance of patients with relatively mild OCD symptoms did not differ significantly from that of patients with severe symptoms.

**Conclusions:** These results do not seem to support the view that impaired executive functioning represents a basic cognitive and pathophysiological feature of Chinese patients with OCD. However, due to study limitations, additional research is required before this conclusion can be well accepted.

## Introduction

Obsessive-compulsive disorder (OCD) is a psychiatric disorder characterized by recurrent obsessions and/or compulsions. Obsessions consist of intrusive repetitive thoughts, images, or impulses. Compulsions are purposeful, repetitive overt or covert behaviors or rituals that are performed by afflicted persons in an effort to relieve anxiety and distress (1). OCD has a lifetime prevalence of 1–3% and is equally common among women and men (2). Patients with OCD often experience a lower quality of life and impaired social and occupational functioning (3, 4).

Studies have shown that patients with OCD typically perform more poorly than matched healthy control participants (HCs) on neuropsychological tests involving high-level cognitive or ‘executive' functions, including planning, working memory, cognitive flexibility, and inhibitory motor control (5–7). These results have led to the hypothesis that impaired executive functioning (EF), including hyper excitability of the orbital frontal cortex and its functional connections, is a core cognitive and pathophysiological feature of OCD (8, 9). Indeed, the current dominant view on the neuropathology of OCD focuses on abnormalities in prefrontal-striatal circuits implicated in EF (10). EF refers to various general-purpose cognitive-control abilities, mainly supported by the prefrontal cortex (PFC), that allow individuals to regulate their thoughts and behaviors (11). EF deficits thus have important consequences for daily-life functioning and may be major contributors to the lack of cognitive flexibility and the perseverative, repetitive behaviors that are cardinal symptoms of OCD (12). Unfortunately, cognitive studies of patients with OCD have not always yielded consistent findings (12, 13). This makes it difficult to arrive at a clear picture of the cognitive functions that are impaired and those functions that are not impaired in patients with OCD. Gaining such understanding could help clinicians to target psychological interventions for OCD according to the integrity of the patients' cognitive functioning.

Meta-analytical reviews of the literature have identified various factors that probably contributed to the mixed findings of studies examining cognitive functioning in adult patients with OCD (12–14),One important factor is the type of cognitive task used to assess the patients' cognitive functioning, which varied greatly between studies (13). Another potentially important source of between-study variability in results is the size and nature of the patient sample examined, including clinical characteristics (e.g., symptom severity, medication status, presence of psychiatric comorbidities) and demographics (e.g., age, sex, intelligence). In this context, it should also be noted that the currently available data concerning cognitive function in OCD primarily come from Western countries and cultures, which raises the question whether the study results can be generalized to non-Western patient populations.

Against this background, the present study was designed to assess cognitive functioning in Chinese patients with OCD and HCs. Based on previous neuropsychological and neuroimaging studies of patients with OCD (8), we focused on several cognitive functions within the broad domain of executive functioning, particularly response inhibition, spatial working memory, planning, and cognitive flexibility. The study objective was twofold: (1) to evaluate several executive functions in Chinese patients with OCD and HCs along with testing their basic learning and memory, and (2) to evaluate differences in executive functions between patients classified as having either relatively mild or severe OCD symptoms. Our main hypothesis was that the patients with OCD, especially those with severe symptoms, would be characterized by impaired executive functions. To assess participants' cognitive functioning, we used several tests of the Cambridge Neuropsychological Test Automated Battery (CANTAB), which is a widely used, validated, and standardized neurocognitive test battery (15). The CANTAB is non-verbal in nature, non-sensitive to gender, and principally culture-free (16), which makes this instrument well-suited for the purpose of the present study.

## Materials and Methods

### Participants

**Figure 1:**
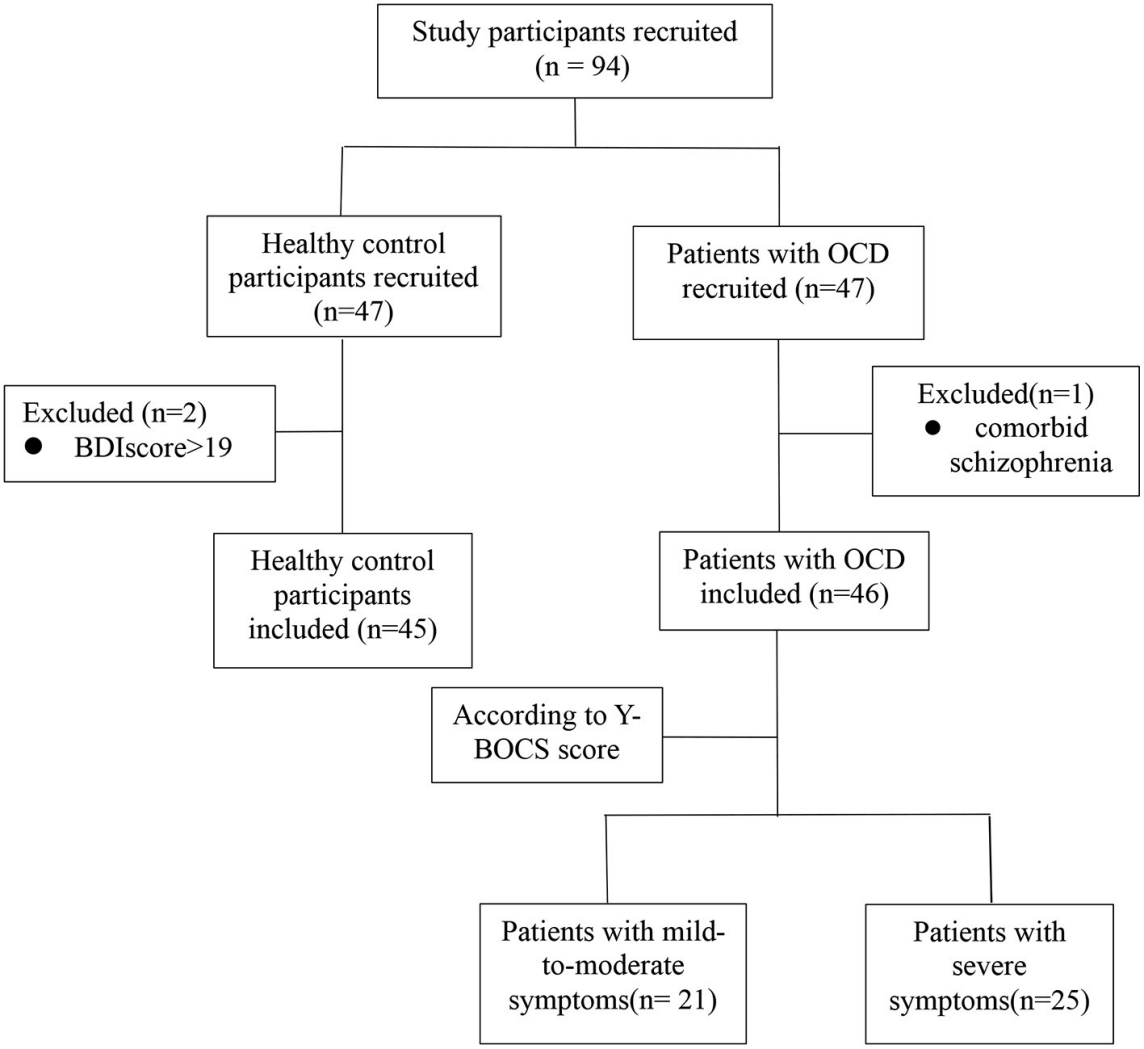
Flowchart illustrating the exclusion, inclusion, and classification of study participants.

Forty-seven patients with OCD (diagnosed by an expert psychiatrist using clinical interview and WHO ICD-10 criteria) were recruited from the Department of Functional Neurosurgery of Ruijin Hospital and the Department of Psychological Medicine of Zhongshan Hospital over a 29-month period (Jun 30, 2018–Nov 25, 2020). Forty-seven HCs were recruited from the community by means of local advertisements. Patients and HCs all lived in Shanghai or the surrounding area. For both patients and HCs, we only included participants ranging in age from 18 to 65 years. Exclusion criteria for patients were as follows: suspected or diagnosed intellectual disability and presence of lifetime neurologic disease/brain trauma, hypothyroidism/hyperthyroidism, or any other clinical conditions that may influence the validity of neuropsychological assessment. Additionally, patients were included only if diagnosed with OCD while having no comorbid psychotic disorder (e.g., schizophrenia) and no major physical comorbidities. The presence of a comorbid anxiety or mood disorder did not constitute an exclusion criterion. One patient was found to have comorbid schizophrenia and was excluded from the study. HCs were included only if they reported to have no lifetime history of psychiatric disorders. They were further screened for symptoms of depression, using the Beck Depression Inventory (BDI), and those with a BDI score of more than 19 were excluded (two participants). Thus, 46 patients with OCD and 45 HCs were included in this study. Figure 1 illustrates the exclusion, inclusion, and classification of study participants. The study was performed in accordance with the Declaration of Helsinki and approved by the ethics committees of Ruijin Hospital affiliated with Shanghai Jiao Tong University School of Medicine and Zhongshan Hospital affiliated with Fudan University. All participants provided written informed consent.

### Clinical Symptom Assessment

The severity of the patients' OCD symptoms was assessed by expert psychiatrists/clinical psychologists who were blinded to the patients' CANTAB results while employing the Chinese version of the Yale-Brown Obsessive-Compulsive Symptom Checklist (Y-BOCS) (17). We used the total score on the Y-BOCS (ranging from 0 to 40), along with the separate subscale scores for obsessions (0–20) and compulsions (0–20), to categorize the severity of the patients' OCD symptoms as follows: “mildly severe” [total score, 6–15 (*n* = 4) or subscale scores, 6–9 for either obsessions or compulsions]; “moderately severe” [total score, 16–25 (*n* = 16) or subscale scores, 10–14 for either obsessions or compulsions (*n* = 1)]; and “severe” [total score, >25 (*n* = 17), or subscale scores, 15 or higher for either obsessions (*n* = 8) or compulsions] (18, 19). Because the number of patients categorized as having “mildly severe” OCD symptoms was relatively low, precluding statistical analysis, we collapsed the “mildly severe” and “moderately severe” categories into one symptom category. Accordingly, the OCD group was divided into one subgroup of patients with relatively mild-to-moderate symptoms (*n* = 21) and another subgroup of patients with severe symptoms (*n* = 25). At the time of enrolment, all patients with OCD were taking selective serotonin reuptake inhibitors (SSRIs), except for four patients in the mild-to-moderate group and six patients in the severe group, who were taking no medication.

### Neuropsychological Assessment

The CANTAB (CANTAB Connect Research) was administered to each participant in a quiet hospital room by a psychologist who had received intensive training in its administration. Participants had to indicate their responses to the information in the computerized cognitive tests by touching a screen (iPad 6 MRJN2CH/A, Apple, CA, USA). We focused on testing the domain of executive functioning, in particular response inhibition, spatial working memory, planning, and attentional set shifting, along with testing new associative learning and visual recognition memory. Because not all participants were able to proceed to the next stage in each test, the number of participants yielding data for statistical analysis differed by test (Tables 1A,B).

**Table 1A:** Number of study participants with incomplete performance data as a function of group and cognitive test.

**Test**	**Number of Patients**		**Number of HCs**	
	**Incomplete test data**	**Included in** **analyses**	**Incomplete test data**	**Included in** **analyses**
SST	3	43	0	45
SWM	0	46	0	45
PAL	0	46	0	45
SOC	2	44	11	34
IED	2	44	11	34

*HCs, healthy control participants; IED, intra-/extra-dimensional set shifting; PAL, paired associates learning; SOC, stockings of Cambridge; SST, stop signal task; SWM, spatial working memory*.

**Table 1B:** Number of patients with incomplete performance data as a function of symptom severity.

**Test**	**Number of patients with mild-to-moderate OCD symptoms**		**Number of patients with severe OCD symptoms**	
	**Incomplete test data**	**Included in** **analyses**	**Incomplete test data**	**Included in** **analyses**
SST	0	21	2	23
SWM	0	21	0	25
PAL	0	21	0	25
SOC	0	21	2	23
IED	0	21	2	23

*OCD, obsessive-compulsive disorder; IED, intra-/extra-dimensional set shifting; PAL, paired associates learning; SOC, stockings of Cambridge; SST, stop signal task; SWM, spatial working memory*.

#### Stop Signal Task (SST)

The SST is a choice reaction-time task purported to assess response inhibition (20). In this task, participants were required to respond (using their indexes fingers or thumbs) to an arrow (“go“ signal) presented on the screen, which pointed to either the left or right. They were instructed to touch, as quickly as possible, the left side of the screen when the arrow pointed to the left and to press the right side when the arrow pointed to the right. They completed one block of 16 practice trials. Subsequently, participants performed the same task except that they had to withhold their behavioral response when an auditory (“stop“) signal (a beep) was presented. The auditory stop signal was delivered at variable intervals (referred to as the stop-signal delay; SSD) after the presentation of the arrow. The stop-signal RT (SSRT), mean RT on go trials, the mean number of direction errors on go and stop trials, and the SSD time) served as the dependent variables.

#### Spatial Working Memory (SWM)

The SWM task measures the capacity to retain and manipulate spatial information for performing the task at hand. In this task, participants were presented with multiple boxes in an increasing order on the screen, with each box revealing a token after being tapped on. All tokens were dropped in a column, and participants were instructed to avoid the box where they had previously found a token. The main dependent variable was the total number of errors made by participants, that is, the errors associated with returning to the box where a token was previously found (21).

#### Paired Associates Learning (PAL)

The PAL test, involving new associative learning and visual recognition memory, required participants to recall a location previously paired with an object. In this task, they were presented with a set of boxes on the screen, which automatically opened and revealed an object/pattern. The patterns emerging from the boxes during the task were different and occurred one at a time in a randomized order. Subsequently, each of the patterns was displayed one at a time on the center of the screen, and participants were asked to identify the box previously associated with the pattern. The dependent variables consisted of the total number of patterns reached, the total number of attempts, and the total errors adjusted.

#### Stockings of Cambridge (SOC)

The SOC test evaluates planning, that is, the ability to cognitively select an adequate action to reach a desired goal. The participants were shown two images stacked row-wise, where the top image had three stockings suspending three colored balls. The participants were instructed to move the balls in the bottom image in order to replicate the top pattern. The balls could be moved only one at a time and were accompanied by a maximum number of allowed moves. The dependent variables were the number of SOC problems that participants successfully completed in the minimum possible number of moves, and the mean number of moves they required to complete 5-move SOC problems.

#### Intra-Extra Dimensional Set Shift (IED)

The IED test assesses rule acquisition and reversal involving visual discrimination and attentional set shifting. In this task, participants were required to evaluate visual stimuli along one or two physical dimensions (form and color) and to use feedback in order to discover a rule that determined which stimulus was correct. After six correct responses, the rule and/or stimuli changed. Initially, participants could distinguish the visual stimuli easily on the basis of one relevant dimension and the subsequent shifts in rule were intra-dimensional. Next, the visual stimuli could be distinguished only on the basis of a combination of the two stimulus dimensions and the shifts in rule were extra-dimensional. There were nine stages to be completed in the task, with intra- and extra-dimensional rule shifts linked to attentional set shifting occurring at stages 6 and 8, respectively. The dependent variables comprised the number of errors made at stages 4, 6, and 8, as well as the number of stimulus trials completed successfully.

### Statistical Analysis

Initially, we evaluated whether the continuous dependent variables were normally distributed. If this requirement was met, we performed independent-sample *t*-tests to assess mean differences between the patients and HCs and, within the patient group, between patients with mild-to-moderate symptoms and patients with relatively severe symptoms. We conducted Mann–Whitney *U* tests if the normality requirement was not met, and for analyzing differences in the proportion of males and females between groups. Because the CANTAB yielded 15 cognitive performance measures (5 from SST, 1 from SWM, 3 from PAL, 2 from SOC, 4 from IED), a Bonferroni-adjusted level of significance of 0.003 (*p* = 0.05/15 = 0.003, two-tailed) was used to protect against inflated Type I error rates (false positives) due to multiple testing. SPSS v26.0 (IBM, Armonk, NY) was used to analyze the data. The data are available upon request from the corresponding author.

## Results

There were no significant group differences seen between the patients with OCD and HCs in relation to age (*p* = 0.107), sex (*p* = 0.604), and education (*p* = 0.634) (Table 2A). Also, no significant differences were observed between patients with mild-to-moderate symptoms and patients with severe symptoms in age (*p* = 0.149), sex (*p* = 0.156), education (*p* = 0.364), and illness duration (*p* = 0.947) (Table 2B).

**Table 2A:** Clinical and demographic characteristics of study participants.

		**Patients(n=46)**		**HCs (n=45)**		**Between-group comparison**	
		***M (SD)***	**Range**	***M (SD)***	**Range**	**Test statistics**	***P***
Age (years)		32.7 (8.5)	19–56	35.2 (6.5)	21–56	*t* = 1.63	0.107
Sex (male/female)		25/21		22/23		*Z* = −0.52	0.604
Education (years)		13.6 (3.6)	5–22	14.1 (3.1)	5–19	*Z* = −0.48	0.634
Illness duration (years)		11.3 (7.6)	1–36				
Y-BOCS score	Obsession	13.5 (5.1)	0–20				
	Compulsion	10.0 (6.4)	0–20				
	Total score	23.5(8.8)	4–38				

*HCs, healthy control participants; M, mean; SD, standard deviation; Y-BOCS, Yale-Brown Obsessive-Compulsive Symptom Checklist*.

**Table 2B:** Clinical and demographic characteristics of patients classified according to symptom severity.

		**Patients with mild-to-moderate OCD symptoms (** ***n*** **=** **21)**		**Patients with severe OCD symptoms (** ***n*** **=** **25)**		**Between-group comparison**	
		***M (SD)***	**Range**	***M (SD)***	**Range**	**Test statistic**	***P***
Age (years)		34.7 (9.3)	21–56	31 (7.6)	19–49	*t* =1.47	0.149
Sex (male/female)		9/12		16/9		*Z* = 1.42	0.156
Education (years)		14.3 (3.5)	8–22	13 (3.6)	5–18	*Z* = −0.91	0.364
Illness Duration (years)		11.2 (7.2)	2–29	11.4 (8.1)	1–36	*t*= −0.09	0.927
Y-BOCS (subscale and scale score)	Obsession	9.1 (3.9)	0–16	17.1 (2.3)	13–20	*Z*= −5.46	<0.001
	Compulsion	8.6 (3.2)	0–13	11.2 (8)	0–20	*Z* = −2.14	0.032
	Total score	17.7 (5.8)	4–25	28.3 (8.1)	15–38	*t* = −4.99	<0.001

*OCD, obsessive-compulsive disorder; M, mean; SD, standard deviation; Y-BOCS, Yale-Brown Obsessive-Compulsive Symptom Checklist*.

Table 3 presents the performance data derived from the SST, SWM, PAL, SOC, and IED separately for patients and HCs along with the results of the statistical analysis. Table 4 summarizes the performance data obtained from the patients classified by OCD symptom severity. No significant patient-control differences were observed in any of the cognitive performance measures (all *p* > 0.003) (Table 3). Similarly, cognitive performance of patients with relatively mild-to-moderate OCD symptoms did not differ significantly from the performance of patients with severe symptoms (all *p* > 0.003) (Table 4).

**Table 3:** Cognitive performance data as a function of group and test along with results of between-group analysis^*^.

**Test**	**Performance measure**	**Patients**	**HCs**	**Test statistics**	***P***
SST	Stop Signal Reaction Time (ms)	238.3 (35.7)	269.8 (69.2)	*Z*= −1.95	0.052
	SST Median RT (ms)	513.8 (67.1)	496.7 (58.0)	*t* = −1.28	0.204
	SST Direction Errors: Go Trials	1.6 (2.6)	2.1 (3.2)	*Z* = −0.67	0.501
	SST Direction Errors: Stop Trials	41.5 (3.3)	43.3 (3.9)	*Z*= −2.55	0.011
	Stop signal delay (ms)	256 (96.5)	231.5 (65.2)	*t* = −1.41	0.161
SWM	Total Errors	8.5 (7.9)	11.3 (8.1)	*Z* = −1.67	0.096
PAL	Number of Patterns Reached	7.8 (0.6)	8.1 (1.3)	*Z* = −1.41	0.159
	Total Attempts	7.4 (2.1)	7.8 (2.0)	*Z* = −1.09	0.274
	Total Errors (Adjusted)	15.4 (12.8)	14.4 (10.8)	*Z* = −0.12	0.902
SOC	Problems Solved in Minimum Moves Total (all moves)	8.2 (2.3)	7.4 (2.3)	*Z*= −1.43	0.153
	Mean Moves (five Moves)	6.9 (1.7)	7.2 (1.6)	*t*= −0.86	0.392
IED	Errors (Stage 4)	0.7 (2.4)	1.1(3.9)	*Z* = −1.36	0.174
	Errors (Stage 6)	1.9 (3.9)	4.9(8.8)	*Z* = −0.65	0.516
	Errors (Stage 8)	9.3 (10.2)	12.4 (11.0)	*Z* = −1.29	0.197
	Stages Completed	8.2 (1.5)	7.6 (2.1)	*Z* = −1.52	0.128

*HCs, healthy control participants; IED, intra-/extra-dimensional set shifting; PAL, paired associates learning; SOC, stockings of Cambridge; SST= stop signal task; SWM, spatial working memory.*

^*^Data values represent group means and standard deviations.

**Table 4:** Performance data as a function of patient subgroup and cognitive test along with results of between-subgroup analysis^*^.

**Test**	**Performance measure**	**Patients with mild-to-moderate OCD symptoms**	**Patients with severe OCD symptoms**	**Test statistic**	***P***
SST	Stop Signal Reaction Time (ms)	241.9 (32.9)	235.2 (38.3)	*t* = 0.61	0.544
	SST Median RT (ms)	524.9 (66.6)	503.3 (67.4)	*t* = 1.06	0.296
	SST Direction Errors: Go Trials	0.9 (1.6)	2.3 (3.2)	*Z* = −1.55	0.121
	SST Direction Errors: Stop Trials	40.7 (3.2)	42.3 (3.3)	*t* = - 1.62	0.113
	Stop signal delay (ms)	284.2 (68.8)	232.3 (110.6)	*t =* 1.86	0.069
SWM	Total Errors	9.3 (7.6)	7.9 (8.2)	*Z* = −0.72	0.469
PAL	Number of Patterns Reached	7.8 (0.6)	7.8 (0.7)	*Z* = −0.27	0.790
	Total Attempts	8.0 (2.4)	6.9 (1.7)	*Z =* −1.43	0.153
	Total Errors (Adjusted)	17.5 (14.0)	13.7 (11.6)	*t*= −0.99	0.326
SOC	Problems Solved in Minimum Moves Total (all moves)	7.9 (2.7)	8.5 (2.0)	*t* = −0.87	0.387
	Mean Moves(five Moves)	7.4 (2.0)	6.4 (1.2)	*t*= 2.05	0.047
IED	Errors (Stage 4)	0.9 (3.2)	0.5 (1.2)	*Z* = −0.98	0.326
	Errors (Stage 6)	0.9 (0.8)	2.8 (5.2)	*Z* = −1.65	0.099
	Errors (Stage 8)	12.8 (11.1)	6.1 (8.1)	*Z* = −1.79	0.074
	Stages Completed	8.1 (1.3)	8.3 (1.7)	*Z* = −1.05	0.292

*OCD, obsessive-compulsive disorder; IED, intra-/extra-dimensional set shifting; PAL, paired associates learning; SOC, stockings of Cambridge; SST = stop signal task; SWM, spatial working memory.*

^*^Data values represent group means and standard deviations.

## Discussion and Conclusion

In this study, we employed the CANTAB to evaluate several executive functions in Chinese patients with OCD and HCs. No significant patient-control differences were observed in the performance of tests of response inhibition, spatial working memory, planning, and set shifting. In addition, the two groups displayed no significant differences in cognitive performance involving basic learning and memory. Moreover, within the patient group, no significant performance differences were detected between patients who were classified as having either relatively mild or severe OCD symptoms. These results are unexpected and do not seem to support the view that impaired executive functioning is a core cognitive and pathophysiological feature of OCD (5–9). However, several factors partly related to limitations of the present study need to be considered before this conclusion can be well accepted.

First, our patient group was comparable to the HC group with respect to age, sex, and education, but it remains possible that preexisting group differences in other variables relevant to cognitive performance, such as socioeconomic status, medication status, or intelligence, contributed to the present results. For example, our OCD group mainly consisted of patients who were taking SSRIs at the time of testing, which may have improved their cognitive performance (22). Yet, medication status cannot easily explain the nonsignificant differences between patients with relatively mild and severe OCD symptoms because most patients in both subgroups were taking SSRIs at the time of testing.

Second, although the present study was not a cross-cultural study and the CANTAB is presumed to be culturally independent, it is possible that cultural factors contributed to the present results, precluding a direct comparison with prior findings from studies conducted in Western societies and patient populations. Therefore, it remains to be determined whether the present findings can be generalized to patients with OCD in other cultures and societies. Third, we employed the Bonferroni correction, which is an adequate but conservative method for controlling Type I errors (false positive findings) due to multiple testing. Accordingly, the use of this method may have controlled Type I errors but at the cost of increasing Type II errors (false negatives) and hence, may have reduced the statistical power of the study to detect small but true patient-control differences in cognitive performance. Indeed, meta-analytical reviews of the literature indicate that patient-control differences in cognitive performance are generally modest, with effect sizes ranging from small to medium for tests of EF (12, 13). Similarly, within a given cognitive task, effect sizes may differ across the dependent variables used for analyzing performance differences [e.g., for SST, a large effect size has been found for SSRT but only a small and nonsignificant effect for performance accuracy (14)].

However, due to some limitations of this study, these results should be interpreted with caution. The first limitation of the present study concerns the small sample sizes examined, which seem to be insufficient to reliably detect cognitive deficits in patients with OCD. Secondly, influenced by the sample size, we did not make a detailed division according to the symptoms of OCD for cognitive comparison. Thirdly, more research and cross-cultural studies are needed to determine whether these results can be replicated in another sample of Chinese patients and whether they can truly be generalized to patients living in other countries and sociocultural cultures.

In conclusion, we observed no significant differences between Chinese patients with OCD and healthy community volunteers in cognitive tests assessing executive functions. However, due to study limitations, additional cognitive studies including large, well-characterized samples of Chinese patients with OCD and matched HCs, as well as cross-cultural studies, are needed to substantiate or qualify the present findings.

## Data Availability Statement

The raw data supporting the conclusions of this article will be made available by the authors, without undue reservation.

## Ethics Statement

The studies involving human participants were reviewed and approved by Ruijin Hospital and Zhongshan Hospital. The patients/participants provided their written informed consent to participate in this study.

## Author Contributions

ZZ, WL, and CZ were responsible for the study concept and design. HL and RC contributed to the acquisition of behavioral data. JH and NZ assisted with data analysis and interpretation of findings. HR and JH drafted the manuscript. ZZ and WL provided critical revision of the manuscript for important intellectual content. All authors critically reviewed content and approved the final version for publication.

## Conflict of Interest

The authors declare that the research was conducted in the absence of any commercial or financial relationships that could be construed as a potential conflict of interest.

## Publisher's Note

All claims expressed in this article are solely those of the authors and do not necessarily represent those of their affiliated organizations, or those of the publisher, the editors and the reviewers. Any product that may be evaluated in this article, or claim that may be made by its manufacturer, is not guaranteed or endorsed by the publisher.
