# Supplementary material for: Executive Functioning in Chinese Patients With Obsessive Compulsive Disorder
Source: Front Psychiatry. 2021 Aug 25;12:662449. doi: 10.3389/fpsyt.2021.662449 (PMC8424121; doi:10.3389/fpsyt.2021.662449)
Supplement: Supplementary file 1 [file Image_1.pdf]

**Supplementary Figure 1.**

**The boxplot (separated by group) for each of the variables used in the study**

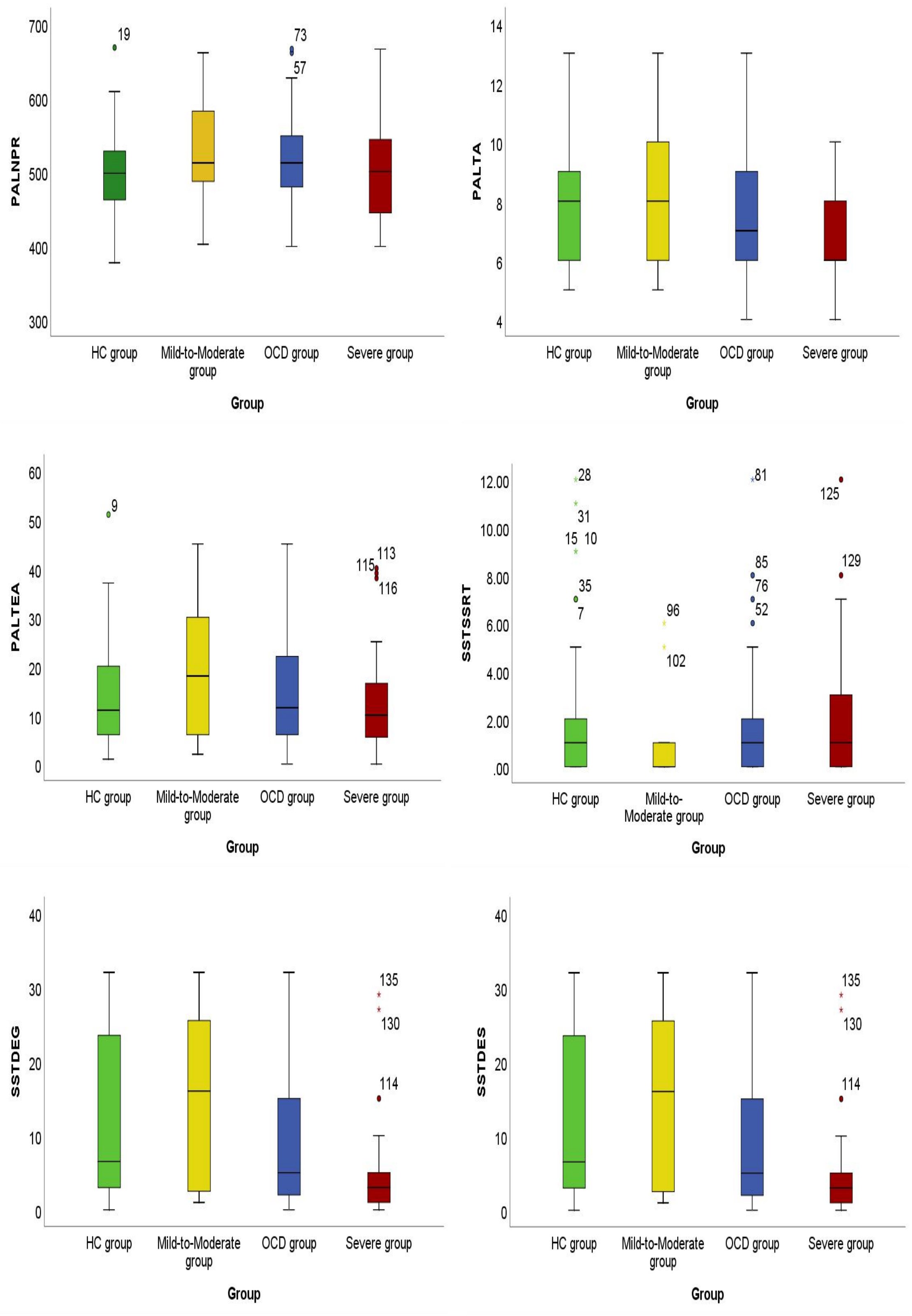

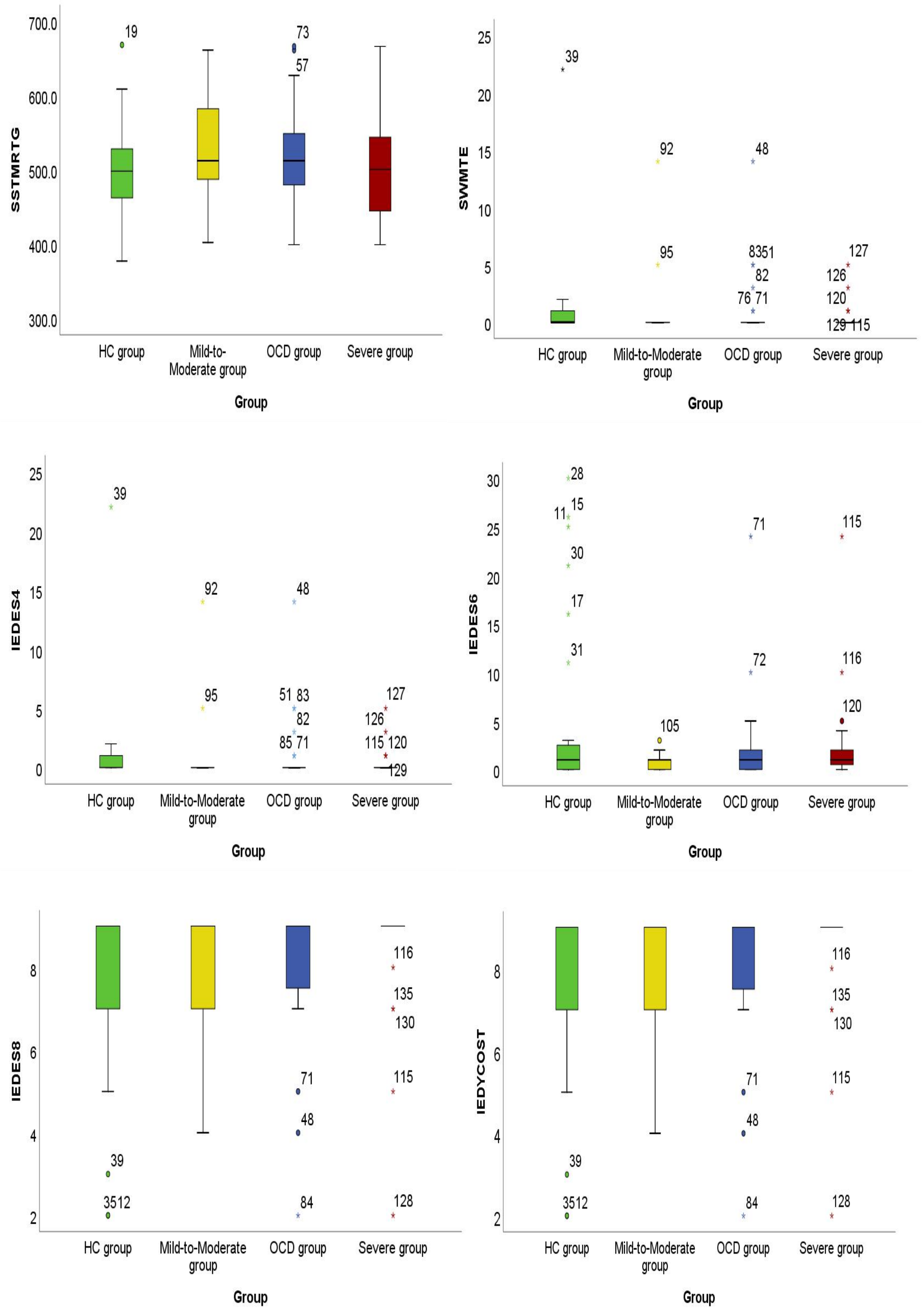
SSTMRTG
700.0
600.0
500.0
400.0
300.0
19
73
57
HC group
Mild-to-Moderate group
OCD group
Severe group
Group
SWMTE
25
20
15
10
5
0
39
92
48
95
8351
82
76 71
127
126
120
129 115
HC group
Mild-to-Moderate group
OCD group
Severe group
Group
IEDES4
39
92
48
95
51 83
82
85 71
127
126
115 120
129
HC group
Mild-to-Moderate group
OCD group
Severe group
Group
IEDES6
30
25
20
15
10
5
0
28
15
11
30
17
31
105
71
72
115
116
120
HC group
Mild-to-Moderate group
OCD group
Severe group
Group
IEDES8
8
6
4
2
39
3512
71
48
84
116
135
130
115
128
HC group
Mild-to-Moderate group
OCD group
Severe group
Group
IEDYCOST
39
3512
71
48
84
116
135
130
115
128
HC group
Mild-to-Moderate group
OCD group
Severe group
Group

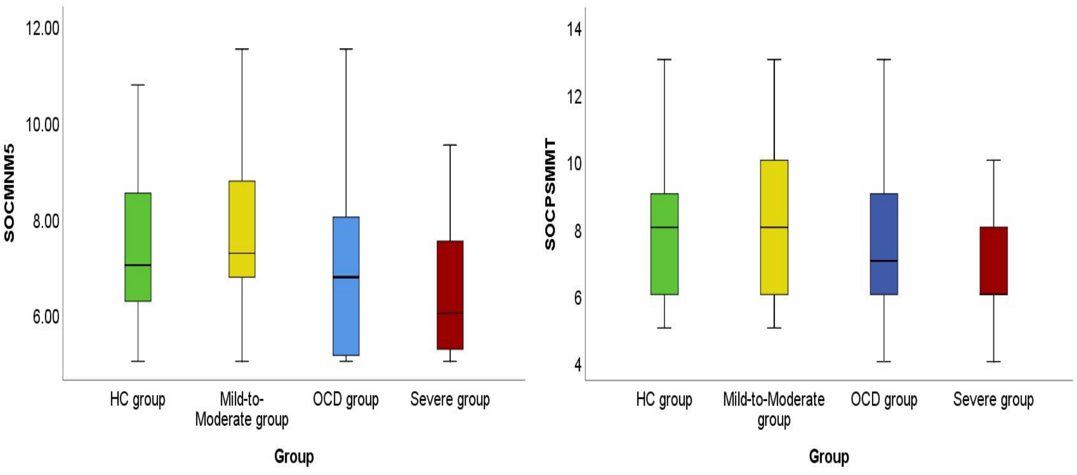
SOCMNM5
12.00
10.00
8.00
6.00
HC group
Mild-to-Moderate group
OCD group
Severe group
Group
SOCPSMMT
14
12
10
8
6
4
HC group
Mild-to-Moderate group
OCD group
Severe group
Group
